# Cholesterol-Lowering Drugs on Akt Signaling for Prevention of Tumorigenesis

**DOI:** 10.3389/fgene.2021.724149

**Published:** 2021-09-16

**Authors:** Navneet Kumar, Chandi C. Mandal

**Affiliations:** ^1^Department of Biochemistry, All India Institute of Medical Sciences, Bhopal, India; ^2^Department of Biochemistry, School of Life Sciences, Central University of Rajasthan, Ajmer, India

**Keywords:** cholesterol, cholesterol-lowering drugs, Akt signaling, cancer, statins, fenofibrate

## Abstract

Cholesterol has been reported to be accumulated in cancer cells. The metabolic dysregulation of the cholesterol is associated with tumor development and progression. The cholesterol-lowering drugs have been found to be involved in the prevention and treatment of various cancers. Akt, a serine/threonine kinase, can modulate the role of several downstream proteins involved in cell proliferation, migration, invasion, metabolism, and apoptosis. Since its involvement in several signaling pathways, its dysregulation is commonly reported in several cancers. Thus, targeting Akt could be an effective approach for cancer prevention and therapy. Cholesterol-lowering drugs have been found to affect the expression of Akt, and its activation in the cancer cells and thus have shown anticancer activity in different type of cancers. These drugs act on various signaling pathways such as PTEN/Akt, PI3k/Akt, Akt/NF-κB, Akt/FOXO1, Akt/mTOR, etc., which will be discussed in this article. This review article will discuss the significance of cholesterol in cancer cells, cholesterol-lowering drugs, the role of Akt in cancer cells, and the effects of cholesterol-lowering drugs on Akt in the prevention of therapy resistance and metastasis.

## Introduction

Cholesterol, a 27-carbon molecule, is distributed throughout the human body and shows a vital role in the cell membrane, nerve conduction, steroid hormone synthesis, vitamin D synthesis, and many more. Diet is having a significant amount of cholesterol which is absorbed in the gastrointestinal tract with the help of bile salts and the involvement of NPC1L1 (a cholesterol transporter), NUMB (a clathrin adaptor), and LIMA1 (an adaptor protein) (Altmann et al., 2004; Goldstein and Brown, 2009; Li et al., 2014). It can also be synthesized *de novo* by almost all cells of the body. Beyond *de novo* synthesis, cells also take up the cholesterol from low-density lipoprotein (LDL) present in the circulation by LDL-receptor mediated endocytosis (Goldstein and Brown, 2009). A balance between cholesterol synthesis and intestinal absorption is critically important for maintaining the cholesterol level in the body. Excess cholesterol is balanced by reverse cholesterol transport to the liver for biliary elimination (Tall et al., 2001; Brewer and Santamarina-Fojo, 2003). Transcriptional regulator sterol regulatory element-binding protein-2 (SREBP-2) and liver X receptors are the critical regulators for maintaining cholesterol homeostasis. The cholesterol homeostasis is maintained by the cholesterol level in the endoplasmic reticulum. When cholesterol level is low in the endoplasmic reticulum, SREBP-2 translocates to the nucleus from the endoplasmic reticulum via Golgi. It leads to the expression of genes responsible for cholesterol synthesis and uptake from outside (Ikonen, 2008). Excess of cholesterol, termed hypercholesterolemia, leads to atherosclerosis, where arteries become narrow down and then blocked, leading to slowing down or blocking of the blood flow to vital organs. It has also been reported that elevated cholesterol also deteriorates bone health (Mandal, 2015).

## Cholesterol in Cancer Cells

In 1909, it was first observed that cholesterol is deposited in the cells of the malignant tissues (White, 1909). A number of epidemiological and basic studies have shown the association between high cholesterol and increased risk of cancer (Mandal and Rahman, 2014; Mandal, 2015; Mandal et al., 2016). It is now known that cholesterol helps cancer cells in proliferation, migration, invasion and epithelial to mesenchymal transition (EMT) (Liu Z. et al., 2018; Sharma et al., 2019; Wang et al., 2019; Huang et al., 2020; Bandyopadhayaya et al., 2021; Patel and Kashfi, 2021). Highly proliferating cancer cells need a continuous supply of cholesterol for membrane biogenesis and numerous other functions of the cells. Thus, cholesterol import, its biosynthesis, and its export all are modulated in cancer cells. Multiple mechanisms promote deregulation of cholesterol homeostasis and lead to cancer development (Smith and Land, 2012; Yun et al., 2014; Vassilev et al., 2015; Jun et al., 2020; Chan et al., 2021). The transcriptional controller of cholesterol biosynthesis SREBP-1 and 2 are elevated in various cancers (Li et al., 2016; Jie et al., 2019; Sharma et al., 2019). There are several oncogenic signals also which are known to modulate the cholesterol synthesis in cancer cells. Constitutive expression of oncogenic PI3K/Akt pathway is one of them which activate SREBP and increase the synthesis of cholesterol. It also induces LDL-R mediated cholesterol import and inhibits ABCA-1 facilitated cholesterol export (Porstmann et al., 2008; Dong et al., 2014). In hepatocellular carcinoma, Akt-mediated activation of phosphoenolpyruvate carboxykinase 1 (PCK-1) activates SREBP proteins and promotes tumor cell proliferation (Xu et al., 2020). In prostate cancer, Akt leads to elevated intracellular cholesterol levels and encourages cancer aggressiveness and bone metastasis (Thysell et al., 2010; Yue et al., 2014). Akt/mTORC1/SREBP pathway promotes cell growth by elevating cholesterol synthesis (Porstmann et al., 2008). By co-operating at multiple levels, the Hippo and p53 signaling pathways regulate cholesterol levels by controlling SREBP activity (Aylon and Oren, 2016). Mitochondrial cholesterol is reported to be elevated in several cancer types. Its import into mitochondria is regulated by StAR protein (Christenson and Strauss, 2000). In hepatocellular carcinoma, elevated StAR protein was linked with the increased cholesterol levels, and its knockdown effectively augmented the sensitivity of cancer cells toward chemotherapeutic agents (Montero et al., 2008). In colon cancer, ABC1 pump, which is involved in pumping out the cholesterol from mitochondria, expression inhibition by oncogenic mutations or loss of function mutation is associated with raised mitochondrial cholesterol levels. This elevation of cholesterol level leads to inhibition of apoptosis and increases cancer cell survival (Smith and Land, 2012).

Other than cholesterol metabolism deregulation and mitochondrial cholesterol accumulation, cholesterol metabolites are also linked with various cancers. One of the cholesterol metabolites, i.e., steroid hormone estrogen, is established in cancer development (Hsu et al., 2017; Rodriguez et al., 2019). Overall, cholesterol accumulation is one of the hallmarks of cancer development and its progression.

## Cholesterol-Lowering Drugs

Hypercholesterolemia is a condition of too much non-HDL cholesterol in the blood, increasing fat deposits in the arteries, and the risk of blockages. Long-term exposure to hypercholesterolemia can lead to atherosclerosis, resulting in cardiovascular disease (Śliż et al., 2019). Cholesterol-lowering drugs are the established tools to control hypercholesterolemia and cardiovascular disease (Reiner et al., 2011). Several *in vitro*, *in vivo*, and clinical trial studies have shown that these drugs also have promising roles in cancer treatment (Giacomini et al., 2021). The currently used cholesterol-lowering drugs are statins, citrate lyase inhibitors, fibrates, bile acid sequestrants, and selective cholesterol absorption inhibitors (Catapano et al., 2016). Statins are the 3-hydroxy-3-methyl-glutaryl-coenzyme A reductase (HMG-CoA reductase) inhibitors, a key enzyme in synthesizing cholesterol (Alberts, 1988). The approved statins for lowering the cholesterol level by the United States are fungi-derived- lovastatin, pravastatin, simvastatin, and synthetically derived- rosuvastatin, atorvastatin, fluvastatin, pitavastatin. The central role of statins is to lower the LDL-C level, and the maximum efficacy of reducing the LDL-C by 60% has been reported in rosuvastatin (Feingold, 2000). Other than LDL-C, statins can also lower triglyceride and VLDL levels and control hyperlipidemia (Stein et al., 1998; Feingold, 2000). Other than cholesterol-lowering properties, statins show pleiotropic effects also. For example, statins can reduce the C-reactive protein level and offer an anti-inflammatory effect (Joshi and Jacobson, 2010). They also provide anti-proliferative properties, antioxidant properties and attenuate vascular remodeling (Liao, 2005). Several scientific reports have shown the correlation between statins and cancer cells. In cancer cells, statins reduce proliferation, migration, and invasion (Di Bello et al., 2020). They can minimize cancer’s negative consequences and increase the survival time (Gupta et al., 2019). Ezetimibe, a cholesterol absorption inhibitor, impedes cholesterol absorption in the intestine and helps to reduce LDL-C levels (Bruckert et al., 2003). This drug is typically used in combination with statins as it is less effective in lowering the cholesterol level alone (Feingold, 2000). Ezetimibe also shows the anti-tumor effects by inhibiting angiogenesis, as shown for the hepatic tumors of Pten^Δhep^ mice with hypercholesterolemia (Miura et al., 2019). Bempedoic acid, a citrate lyase inhibitor, is another cholesterol-lowering drug and can inhibit adenosine triphosphate-citrate lyase (ACLY), catalyzing the formation of acetyl-CoA in the cytoplasm. It can reduce non-HDL-C, LDL-C, and apolipoprotein B (Laufs et al., 2019). ACLY plays a decisive role in cancer metabolism. It provides the acetyl CoA in lipid synthesis, aspartate production (required for nucleotide), and NADPH production for biosynthesis purposes (Icard et al., 2020). Inhibition of it by bempedoic acid might be crucial to show its anti-cancerous effects. Fenofibrate comes under the fibrate category of cholesterol-lowering drugs, which are used to treat hyperlipidemia and hypercholesterolemia. Fenofibrate activates peroxisome proliferator-activated receptors α (PPARα), a transcription factor that stimulates the beta-oxidation of fatty acids (Schoonjans et al., 1996). It has been found to reduce cellular proliferation and boost apoptosis in cancer cells (Yamasaki et al., 2011; Sun et al., 2019). Since cholesterol-lowering drugs can modulate proliferation, migration, and invasion and inhibit tumorigenesis, this review article will discuss the effects of these drugs in correlation with Akt signaling.

## Akt Signaling and Cancer

Akt, also known as Protein kinase B, is a serine/threonine kinase. On phosphorylation, it converts into Phospho-Akt, an active form of Akt that acts on its downstream targets. There are three isoforms of the Akt gene, i.e., Akt1, Akt2, and Akt3, and principally it is Akt1 isoform (Datta et al., 1999). They differentially act in normal cells and cancer cells (Bellacosa et al., 1993). Once survival factors activate Akt, it translocates to the plasma membrane, gets phosphorylate, and activates the downstream targets. Various growth factors such as insulin-like growth factor, vascular endothelial growth factor, epidermal growth factor, platelet-derived growth factor (PDGF), and other factors such as cytokines, cAMP, hypoxia can induce the kinase activity of the Akt (Datta et al., 1999).

Akt acts as a potential oncogenic molecule and is highly expressed and activated in an extensive range of human cancers (Cerami et al., 2012; Galbraith et al., 2021; Sun et al., 2021). Oncogenic property of Akt by amplification and overexpression is more commonly found in cancers like gastric, glioblastoma, ovarian, breast, pancreatic, prostate, etc. (Staal, 1987; Bellacosa et al., 1995; Cheng et al., 1996; Nakatani et al., 1999; Knobbe and Reifenberger, 2003). Mutation in Akt is not very common as compared to amplification and overexpression. The most frequent Akt mutation is found in its PH (pleckstrin homology) domain identified in cancers such as bladder, lung, pancreatic, endometrial, and urothelial (Malanga et al., 2008; Mohamedali et al., 2008; Shoji et al., 2009; Zilberman et al., 2009; Askham et al., 2010). The oncogenic role of Akt can also be displayed by mutations in upstream/downstream molecules such as PI3K, Ras, PTEN, and p27 (Downward, 2003; Mandal et al., 2012). Regulatory changes by *N*^6^-methyladenosine (m^6^A) methylation can also activate Akt signaling and increase proliferation, progression, migration, and invasion of cancer cells (Liu J. et al., 2018; Li et al., 2020; Shi et al., 2020). Post-translational modifications such as tyrosine phosphorylation, lysine modifications, ubiquitination, sumoylation, and acetylation are also important to hyperactivate Akt in cancer cells which leads to tumorigenesis (Chan et al., 2012, 2014; Han et al., 2018).

Akt is a crucial regulator in signaling pathways for cell survival, insulin signaling, angiogenesis, and tumorigenesis (Revathidevi and Munirajan, 2019). It can up-regulate the cell cycle by promoting PCNA, CDK1, and Telomerase Reverse Transcriptase (Koundouros and Poulogiannis, 2018; Zhang and Hu, 2018). It can activate cellular proliferation by phosphorylating tumor suppressor p21 which can arrest the cell cycle by constraining cyclin-CDK complexes (Zhou et al., 2001). Akt also shows the anti-apoptotic activity by regulating the members of the BCL-2 family of proteins. It can control apoptosis through inhibition of BIM (BCL-2-like protein 11), BAD (BCL-2/Bcl-XL-antagonist, causing cell death), caspase 9, and FoxO1 (forkhead box protein O1) (Sangawa et al., 2014; Kizilboga et al., 2019; Revathidevi and Munirajan, 2019; Wu et al., 2019). Akt exerts its effects on glucose metabolism, which is required for rapidly proliferating cells. It promotes Glut1 and Glut4 to the cell membrane and increases glucose transport (Kohn et al., 1996). It also encourages glycolytic enzymes such as hexokinase, phosphofructokinase (PFK)-1, and PFK-2 (Deprez et al., 1997; Gottlob et al., 2001). These metabolic changes in glucose metabolism endorse cell survival. Akt facilitates all these effects via regulating glycogen synthase kinase – 3 (GSK-3), a key controller for phosphorylation of the glycolytic enzymes (Datta et al., 1999). Lipogenesis is a crucial aspect of cancer cell proliferation and signaling (Koundouros and Poulogiannis, 2020). Activation of Akt promotes lipogenesis by providing metabolic intermediates of carbon for anabolism and reducing equivalents in the form of NADPH (Ward and Thompson, 2012). It increases acetyl-CoA synthesis required for lipogenesis by phosphorylating and activating the ATP citrate lyase (ACLY) (Berwick et al., 2002). Akt is a potential activator of mTORC1, which promotes *de novo* lipogenesis on phosphorylation (Porstmann et al., 2008; Saxton and Sabatini, 2017). SREBP-1 is one of the primary transcriptional regulators for lipogenesis. Akt averts the degradation of mature SREBP-1 and promotes lipogenesis. Since Akt is a vital molecule in cancer and is involved in various oncogenic signaling, this review article emphasizes the connection between cholesterol-lowering drugs and cancer prevention via Akt signaling. However, PI3K/Akt signaling is also crucial for various physiological and pathological functions like bone remodeling, cellular hypertrophy and cell differentiation (Mandal et al., 2009; Das et al., 2012; Ghosh-Choudhury et al., 2013; Mandal et al., 2016).

## Cholesterol-Lowering Drugs on Akt Signaling

Cholesterol-lowering drugs have been stated to show anti-tumorigenic properties. They may act on HMG-CoA reductase and reduce the cholesterol level, which disturbs the cancer cells’ lipid rafts and affects the cells’ signaling (Lee E.J. et al., 2014). These drugs also inhibit various signaling pathways and halt cell proliferation, migration and metastatic activity, and encourage apoptosis in cancer cells (Klawitter et al., 2010; Mehta et al., 2017). One of the important signaling which have been found commonly involved in the mode of action of cholesterol-lowering drugs is Akt signaling (Ghosh-Choudhury et al., 2010; Mandal et al., 2011). This review explores the involvement of Akt signaling as a target of cholesterol-lowering drugs to inhibit different types of cancers.

### Lung Cancer

Lung cancer has the highest incidence worldwide and stands at second for its mortality rate (Globocan, 2020). Non-small-cell lung carcinoma (NSCLC) shares 85% of all lung cancer and is insensitive toward cytotoxic chemotherapy (Chen et al., 2014; Hu et al., 2020). In lung cancer, unusual overexpression or stimulation of Akt has been observed, linked with elevated cancer cell proliferation and survival (Song et al., 2019). Simvastatin has been observed to lessen the expression of p-Akt in NSCLC derived A459 lung cancer cells (Hwang et al., 2011). Akt-mediated survival pathway stimulates survivin synthesis, which may inhibit apoptosis in numerous cancer cell lines (Fornaro et al., 2003; Ohashi et al., 2004). Thus, p-Akt inhibition in A459 cells was found to down-regulate the survivin and increase apoptosis, as supported by the reported cleavage of PARP (Hwang et al., 2011). Simvastatin in blend with non-steroidal anti-inflammatory drug sulindac showed synergistic effects on Akt signaling-dependent down-regulation of survivin and elevation of apoptosis in A549 cells (Kim et al., 2015). Lovastatin also shows anti-tumorigenic activity against lung cancer. In A549 cells, lovastatin selectively inhibited the pro-survival pathway of Akt (Sanli et al., 2011). A549 cells have a point mutation in the K-Ras gene, triggering the PI3K/Akt pathway (Okudela et al., 2004). Activated Akt can activate ACLY by phosphorylating it (Berwick et al., 2002). Growth factors lead to the stimulation of PI3K/Akt, which increases the activity of ACLY via Akt-mediated ACLY phosphorylation. ACLY promotes tumor growth in glycolytic tumors, and its inhibition is responsible for the halt in tumor growth and leads to the differentiation of tumor cells (Hatzivassiliou et al., 2005). Hanai et al. (2012) reported that inhibition of PI3k/Akt along with ACLY inhibition led to the enhanced anti-tumor effects of ACLY inhibition. Lovastatin is reported to show anti-tumor effects by diminishing the activity of ACLY via inhibition of Akt in lung cancer (Hanai et al., 2012). Akt is a well-known facilitator of radiation resistance in several cancer cells (Gupta et al., 2002). In A549 cells, the EGFR-PI3k-Akt pathway activation confers radio-resistance (Toulany et al., 2005; Sanli et al., 2011). Lovastatin effectively inhibited the ionizing radiations-induced Akt activation and stimulated activation of AMPK, which led to apoptosis of cancer cells and radiation sensitization (Sanli et al., 2011). AMPK is an AMP-activated kinase that can dephosphorylate and inhibit Akt (Kim et al., 2009) and work synergistically with lovastatin to radio-sensitize cancer cells. Braf/MEK/ERK1/2 cascade facilitates cell proliferation and apoptosis. This cascade can lead to many cancers, including lung cancer (Smalley and Smalley, 2018; Wang Q. et al., 2018; Zhang et al., 2019). Fluvastatin, a synthetic HMG-CoA reductase inhibitor, suppresses Akt and Braf/MEK/ERK1/2 pathways. This suppression leads to the inhibition of NSCLC by preventing cell growth and promoting apoptosis *in vitro* and *in vivo* (Zhang et al., 2019). These studies show how statins inhibit lung cancer via Akt and how Akt can be a crucial target for chemotherapeutic purposes.

### Breast Cancer

Breast cancer is the most reported cancer worldwide and fifth in all cancer deaths (Globocan, 2020). Women with elevated cholesterol have shown high incidences of breast cancer. The mevalonate pathway is the crucial pathway responsible for cholesterol synthesis (Kitahara et al., 2011). The products of the mevalonate pathway are known to promote proliferation, migration, and differentiation of tumor cells (Dimitroulakos et al., 2006). For example, isoprenoid activates Ras and Rho GTPase prenylation, leading to the stimulation of the PI3K/Akt pathway and promoting tumorigenesis (Schieber and Chandel, 2014). Thus, statins here can act like cancerstatic agents that inhibit the mevalonate pathway and inhibit cancer cell growth (Schointuch et al., 2014; Cardwell et al., 2015). In a study by Beckwitt et al. (2018) Akt phosphorylation was decreased by treatment of atorvastatin in three breast cancer cell lines, i.e., MCF-7 RFP, MDA-MB-231 RFP, and MDA-MB-231 RFP/Ecad, and this inhibition was shown even after stimulation with growth factor EGF. It indicates that atorvastatin can inhibit the phosphorylation of Akt even after growth factor stimulation and impede breast cancer cell growth (Beckwitt et al., 2018). In breast cancer, constitutive expression of MAPK/ERK and PI3K/Akt/mTOR signal pathways are significant events that promote cancer cell growth, survival, and metastasis (Downward, 2003; Engelman, 2009). Simvastatin treatment induces apoptosis, inhibits proliferation, suppresses these two pathways, and shows its anti-tumor effects in breast cancer (Wang et al., 2016). The inhibitory effect of simvastatin on phosphorylation of Akt has been detected in MDA-MB-231 xenograft breast tumor model also which led to the repression of antiapoptotic BCL_XL_ expression and promotion of proapoptotic/antiproliferative proteins (Ghosh-Choudhury et al., 2010). Another Akt pathway, i.e., PTEN/Akt pathway, regulates multiple cellular dysfunctions in breast cancer cells, such as proliferation, metabolism, and genomic stability (Mehta et al., 2017). Atorvastatin has been found to increase the PTEN and decrease p-Akt in breast cancer cells and inhibit the PTEN/Akt pathway. The inhibition of the PTEN/Akt pathway by atorvastatin is reported to happen via increasing the expression of RhoB in breast cancer cells (Ma et al., 2019). RhoB is a Ras family member and regulates actin stress fibers and vesicle trafficking (Prendergast, 2001). In cancer cells, it acts as a tumor suppressor gene as it prevents cell proliferation and migration and encourages apoptosis by impeding PTEN/Akt pathway (Jiang et al., 2004). Dephosphorylation of Akt and increased expression of PTEN by statin treatment is also reported to suppress PI3K/Akt/mTOR pathway in ER-positive (MCF-7, T47D) as well as ER-negative (MDA-MB-231) breast cancer cells (Wang et al., 2016). Simvastatin treatment on Triple-negative breast cancer has shown decreased p-Akt and increased expression of PTEN, which led to reduced cell viability (Park et al., 2013). Lovastatin shows similar effects on p-Akt and PTEN in PTEN-expressing MDA-MB-231 cell lines, as demonstrated by atorvastatin and simvastatin (Klawitter et al., 2010). DJ-1 is an oncogene associated with H-Ras and increases cell proliferation and resistance to cell cycle arrest (Nagakubo et al., 1997). The overexpression of this oncogene is positively linked with p-Akt and poor prognosis of breast cancer (Kim et al., 2005). Klawitter et al. (2010) have shown that lovastatin-led PTEN expression caused the reduction in the expression of DJ-1, which might be a crucial controller of p-Akt expression in lovastatin-treated breast cancer cells. This reduction in DJ-1 caused the reduction in the expression of p-Akt in breast cancer cells and this influence was detected in the downstream of DJ-1/PTEN-regulated Akt pathway also on another important protein NDRG1 which plays a significant role in metastatic tumor progression (Bandyopadhyay et al., 2004; Klawitter et al., 2010). In xenograft tumor study by implanting MDA-MB-231 human breast cancer cell in mice, simvastatin treatment significantly showed the reduced p-Akt and increased PTEN level. This study also showed that simvastatin inhibited the phosphorylation of Akt indirectly also by inhibiting binding of NFκB transcription factor to DNA in the PTEN promoter region and allow the expression of PTEN to inhibit p-Akt (Ghosh-Choudhury et al., 2010). Fenofibrate, a non-statin cholesterol lowering drug is used in the therapy of hyperlipidemia and hypercholesterolemia. It is reported to act on Akt/NF-κB pathway and reduce the phosphorylated Akt and NF-κB p65 in SKBR3 and MDA-MB-231 cells (Sun et al., 2019). Akt/NF-κB pathway in breast cancer blocks the activity of pro-apoptotic genes such as Bok, Bax and BIM (Bentires-Alj et al., 2001; Inta et al., 2006). The effect of statins and fenofibrate on Akt shows that it is an important kinase molecule which on modulation can reduce the breast cancer tumorigenesis.

### Colorectal Cancer

Colorectal cancer is the third most common cancer worldwide and the second most common cause of mortality in cancer (Globocan, 2020). The importance of Akt signaling has also been identified in suppressing colorectal cancer by cholesterol-lowering drugs. Mantha et al. (2005) showed the enhanced anti-phosphorylation effects on Akt in colon cancer cell lines by combining lovastatin with gefitinib treatment. Xiao et al. (2008) found the synergistic effects of atorvastatin and celecoxib to inhibit the Akt activation in HCT116 and HT29 cells. It showed that celecoxib alone was required in a high concentration for modulating Akt, but the combination with atorvastatin reduced the required dose of celecoxib and increased the efficacy to inhibit p-Akt. This inhibition of Akt activation led to the modulation of crucial components of the Akt pathway such as PDK1, PI3K, and PTEN and showed anti-cancerous effects (Xiao et al., 2008). It is well known now that statins inhibit the phosphorylation of Akt in cancer cells and show anti-tumorigenic effects (Roudier et al., 2006; Klawitter et al., 2010; Huang et al., 2015). However, simvastatin in HCT116 and HT-29 colon cancer cells has shown elevated levels of p-Akt. This study showed that simvastatin activated Nrf2 (nuclear factor-erythroid 2-related factor 2), which on translocation to the nucleus induced the expression of HO-1 (heme oxygenase-1) related antioxidants via ERK and PI3K/Akt pathway (Jang et al., 2016). Nrf2 is a transcription factor activated in response to cellular stress. It activates the expression of antioxidant defense genes such as HO-1 and glutathione regulatory enzymes in response to ROS (Nguyen et al., 2003; Cuadrado and Rojo, 2008). Statins show their antioxidant and anti-inflammatory effect by inducing the expression of HO-1 and reducing the free radicals formation (Lee et al., 2004; Jang et al., 2016). Although, this study of Jang et al. (2016) could not clear whether the activation of HO-1 by simvastatin protected the cancer cells or reduced the cell proliferation in colon cancer cells. Being the second deadliest cancer worldwide, the role of other cholesterol-lowering drugs on Akt signaling in colorectal cancer is required to explore and understand for better understanding of the mechanism of cholesterol-lowering drugs in the prevention of colorectal cancer.

### Prostate Cancer

It is one of the most common cancer in men worldwide and a prominent cause of death in western countries. Statins have been associated with decreased metastasis and all-cause mortality among prostate cancer (PCa) patients (Lee E.J. et al., 2014; Raval et al., 2016). Akt is an important target molecule in the pathogenesis of PCa (Chen H. et al., 2016). Statins alone, as well as in combination with other drugs, can act on Akt and can inhibit its activation in PCa cells (Rogers et al., 2015; Wang et al., 2017; Sekine et al., 2018). Inhibition of Akt phosphorylation can promote PCa apoptosis and inhibit cell proliferation (Deng et al., 2019). FOXO is a transcription factor regulated by Akt through phosphorylation. It leads to the translocation of FOXO from the nucleus to the cytoplasm and modulates cell survival, growth, and apoptosis (Burgering and Kops, 2002; Lam et al., 2006). Akt/FOXO pathway can regulate cell growth, apoptosis, and survival (Song et al., 2005). Statins such as simvastatin and fluvastatin are reported to significantly reduce the phosphorylation of Akt and FOXO1 (a member of the FOXO family) and promote apoptosis in the PCa cells (Deng et al., 2019). *In vitro* studies in PCa cells show that simvastatin can reduce serum-induced cell migration, colony formation, invasion, and proliferation in prostate cancer. Not only this, it showed the reduced tumor growth in the xenograft model associated with reduced Akt activity (Kochuparambil et al., 2011). A part of PCa cells develops the steroidogenic ability to make androgens from the cholesterol, leading to castration-resistant (CR) prostate cancer. Novel statin derivatives are found to suppress the CR PCa tumorigenicity by inhibiting Akt and androgen receptor pathway. The inhibition of Akt by statins also inhibits the cell migratory ability of PCa, which further supports their ability to inhibit tumorigenicity (Ingersoll et al., 2016). PCa cells contain cholesterol-rich lipid rafts that mediate the constitutive signaling through Akt via the mediation of epidermal growth factor (Zhuang et al., 2002). Statins lead to reduced cholesterol levels and disrupt these lipid rafts, leading to reduced cell survival (Chen X. et al., 2016). These studies indicate that the Akt pathway is one of the crucial targets for statins to mediate tumor suppression in PCa. These studies are further required to extend to other cholesterol-lowering drugs for the future course of PCa treatment.

### Liver Cancer

Liver cancer is the sixth most common cancer worldwide and the third common reason for cancer mortality (Globocan, 2020). A relation between statin treatment, reduction in Akt phosphorylation, and lung cancer reduction have also been established. In HepG2 cells, atorvastatin has been found to inhibit Akt phosphorylation and translocation to the nucleus in mTOR dependent manner. This inhibition of p-Akt prevents the phosphorylation of GSK3β and cell proliferation (Roudier et al., 2006). Statin treatment causes the phosphorylation of mTOR and activates it, which inhibits insulin-induced Akt activation (Pääjärvi et al., 2005; Roudier et al., 2006; Tzatsos and Kandror, 2006). Huang et al. (2015) reported that simvastatin induced cytotoxic effects on HepG2 and Huh7 liver cancer cell lines. They observed that the level of p-Akt was reduced by simvastatin treatment in hepatocellular carcinoma (HCC) cells which were found to be associated with the Notch1 gene (Huang et al., 2015). Notch1 is a member of the Notch family whose role is to regulate growth, apoptosis, migration, and invasion of tumor cells (Ramdass et al., 2007; Wang et al., 2011; Lai et al., 2018). Following the knockout of the Notch1 gene, the simvastatin effect on p-Akt expression was diminished, and the apoptosis of HCC cells was attenuated (Huang et al., 2015). Statins, along with other drugs such as dasatinib (an anticancer drug), NS398 (COX-2 inhibitor), and celecoxib (COX-2 inhibitor), has shown synergistic effects on the inhibition of p-Akt in HCC cells (Gao et al., 2010; Lee S.J. et al., 2014; El Sayed et al., 2018). Other than statins, fenofibrate has shown anti-proliferative effects in Huh7 HCC cells by suppressing the phosphorylation of Akt (Yamasaki et al., 2011). Since p-Akt inhibits localization of p27 in the nucleus (Viglietto et al., 2002), fenofibrate led to the accumulation of p27 in the nucleus by inhibiting phosphorylation of Akt, which led to the cell cycle arrest. Interestingly, the suppression of growth of Huh7 HCC cells was found to be PPARα independent (Yamasaki et al., 2011). Seeing the impact of cholesterol-lowering drugs on the Akt pathway, using them can be an effective strategy to treat/control hepatocellular carcinoma. There is a need to explore the effect of other cholesterol-lowering drugs on Akt and its targets for widening the understanding and new treatment strategies of HCC.

### Pancreatic Cancer

Pancreatic cancer is the twelfth most common cancer worldwide and one of the deadliest human malignancies (WCRF, 2018; Globocan, 2020). Among pancreatic cancer incidences, 96% are exocrine cancer, and pancreatic ductal adenocarcinoma (PDAC) is the predominant one (PCUK, 2018). Pancreatic cancer has a poor prognosis with an overall 5-year survival rate in 5% of affected people (Pourshams et al., 2019). The commonly affected pathways in pancreatic cancer are PI3K/Akt, NF-kB, and MAPK pathways (Altomare et al., 2002; Zhao et al., 2006; Holcomb et al., 2008). Akt pathway is reported to be overexpressed in pancreatic cancer cells and generate resistance against cytotoxic drugs such as gemcitabine (Ng et al., 2000). Thus, this pathway has attracted attention as an effective target to treat pancreatic cancer. Statins have shown the inhibition of pancreatic cancer cells *in vivo* as well as *in vitro*. Atorvastatin is one of the statins reported to decrease p-Akt in Panc-1and MIA PaCa-2 cells and sensitized them toward cytotoxic drugs gemcitabine and 5-Fu. It showed the ability to inhibit the constitutive expression of Akt as well as the insulin-induced expression of Akt in pancreatic cancer cells and thus affected Akt downstream targets. This treatment led to the prevention of cell proliferation and stimulation of cell apoptosis in pancreatic cancer cells (Mistafa and Stenius, 2009). Pancreatic intraepithelial neoplasia (PanIN) is the precursor of PDAC. The progression of PanIN from low-grade PanIN (termed as PanIN-1) to high-grade PanIN (PanIN-2 and -3) and then to ductal adenocarcinoma is believed to be the stages of progression to PDAC (Hruban et al., 2006). PI3/Akt pathway is associated with the expression of several biomarkers involved in the passage from PanIN to PDAC. Atorvastatin inhibits this progression and becomes possible by regulating the PI3/Akt pathway (Mohammed et al., 2012). Akt also impacts the production of acetyl-CoA (Lee J.V. et al., 2014). ACLY is the enzyme involved in generating nucleo-cytosolic acetyl-CoA and is regulated by Akt at the upstream position (Lee J.V. et al., 2014; Carrer and Wellen, 2015). The production and availability of acetyl-CoA highly affect histone acetylation, a dynamic chromatin modification procedure involved in gene regulation (Wellen et al., 2009; Cardwell et al., 2015; Shi and Tu, 2015). In human PDAC, an association has been found between high histone acetylation levels and poor prognosis (Juliano et al., 2016). This Akt-ACLY signaling has been reported to be inhibited by treatment of statins and BET inhibitors which showed suppression of PDAC cell proliferation and tumor growth (Carrer et al., 2019). Overall in pancreatic cancer, statins are effective in impeding cell proliferation and tumor growth by targeting Akt.

### Other Cancers

Oral cancer ranks at the sixteenth position for its incidences as well as mortality in overall cancer worldwide. The study on the effect of cholesterol-lowering drugs on Akt signaling in oral cancer has been minimal and needs to be extended further. One of these studies is by Jan et al. (2016) where fenofibrate was found to reduce Akt and p-Akt levels. Akt can directly communicate with mTOR and activate it, which is mediated by TSC2 and PRAS40 (Gwinn et al., 2008). Thus inhibition of Akt by fenofibrate was associated with elevated expression of TSC2, which antagonized Rheb and reduced mTOR expression. This study did not find any role of PRAS40 and p-PRAS40 in mTOR inhibition by fenofibrate (Jan et al., 2016). Squamous cell carcinoma (SCC) is an epithelial malignancy. It has a limited treatment once converted into a metastatic disease (Greenlee et al., 2000; Breathnach et al., 2001). Receptor tyrosine kinase, mainly EGFR, is associated with the pathogenesis of SCC (Nicholson et al., 2001). Activation of EGFR triggers downstream signaling cascades, such as activation of the PI3K/Akt pathway that regulate cell proliferation and cell survival (Mendelsohn and Baselga, 2000; Dann and Thomas, 2006). Zhao et al. (2010) found that lovastatin can inhibit EGFR, resulting in the inhibition of Akt and its downstream targets in SCC. Statins have also shown anti-leukemic properties, as shown by Vilimanovich et al. (2015) in their studies. High cholesterol levels and metabolism are reported to be important factors for the survival of leukemic cells. Cholesterol synthesis and its import are highly active in these cells (Vitols et al., 1984; Rudling et al., 1998). Statins treatment leads to the reduction in the total cholesterol content of lipid rafts required for maintaining the activity of lipid raft residing Akt (Lasserre et al., 2008). It causes the reduced activation of Akt and its downstream targets mTOR and ribosomal p70S6 kinase in leukemic cells, which otherwise represses the autophagy. Thus, statins can be considered an inducer of autophagy in leukemic cells by inhibiting Akt/mTOR/p70S6K signaling (Vilimanovich et al., 2015). Human acute T lymphocytic leukemia (T-ALL) cells are significantly hampered by fluvastatin and simvastatin. They suppress the T-ALL cell proliferation and promote cell apoptosis by inhibiting the Akt pathway (Wang J.J. et al., 2018). Zeng et al. (2012) showed the effects of simvastatin on human acute monocytic leukemia cell line SHI-1. They showed that simvastatin treatment inhibited cell proliferation and induced apoptosis in SHI-1 cells, which was found to be associated with the changes in the gene expression level of the Akt signaling pathway (Zeng et al., 2012). Cancer cells use glycolysis to generate ATP for fulfilling their energy requirement. Over activated glycolysis pathway generate Methylglyoxal (MG) as a by-product from glyceraldehyde 3-phosphate and dihydroxyacetone phosphate in cancer cells (Phillips and Thornalley, 1993; Richard, 1993). MG is a cytotoxic product that prevents cell proliferation and promotes apoptosis in human leukemia HL-60 cells because of accumulation of MG-DNA adduct accumulation (Kang et al., 1996). Glyoxalase 1 (GLO1) detoxifies MG into D-lactate and protects the cells from damage caused by MG (Santarius et al., 2010). GLO1 is highly expressed in cancer cells (Hooper et al., 1987; Baunacke et al., 2014; Hu et al., 2014). Lovastatin is reported to suppress the expression of GLO1 and HMG-CoA by interrupting the translocation of NF-κB to the nucleus through inhibition of Ras/PI3K/Akt and Ras/Raf/ERK pathway in HL-60 cells (Chen et al., 2015). Cholesterol-lowering drugs have also shown promising effects to inhibit brain tumors via Akt signaling. Gliomas are malignant primary brain tumors resistant to conventional therapies such as radiation and chemotherapy (Wu et al., 2009). Gliomas and other malignant brain tumors also show a high rate of cholesterol synthesis and increased HMG-CoA activity (Grieb et al., 1999). Simvastatin shows anti-proliferative and anti-migration activity and induce apoptosis in U251 and U87 cells in a dose- and time-dependent manner. The modulation of PI3K/Akt/caspase-3 pathway in these cells where p-Akt level was reduced and caspase-3 level was increased led to the induction of apoptosis and showed anti-tumorigenic activity. The reduction in cholesterol content, modification of lipid rafts and translocation of Fas into the lipid rafts was also reported to inhibit U251 and U87 cells (Wu et al., 2009).

## Clinical Relevance of Cholesterol-Lowering Drugs in Cancer

The use of cholesterol-lowering drugs in cancer prevention is well known now. This review article has discussed the significance of cholesterol-lowering drugs in cancer prevention via Akt modulation. Various clinical studies concerning the effects of cholesterol-lowering drugs in cancer treatment are undergoing and need to be completed. There are completed clinical studies also showing how cholesterol-lowering drugs may add a therapeutic approach to cancer treatment. Table 1 shows some of the clinical studies by using different cholesterol-lowering drugs on various types of cancers.

**TABLE 1:** Clinical studies and their outcomes in cancer treatment by using cholesterol-lowering drugs.

Type of cancer	Cholesterol-lowering drug	Clinical trial phase	Clinical trial number	Status	Outcome	Reference
Lung cancer	Simvastatin	Phase II	NCT00452244	Completed	Improved efficacy of gefitinib	Han et al., 2011
Breast cancer	Atorvastatin	Phase II	NCT00816244	Completed	Anti-proliferative	Feldt et al., 2015
	Simvastatin	Phase II	NCT00334542	Completed	Decreased Estrone sulphate	Higgins et al., 2012
Colorectal cancer	Simvastatin	Phase II	NCT02026583	Completed	Shows comparable clinical efficacy along with XELOX and bevacizumab	Kim et al., 2019
		Phase II		Completed	Increased time to progression	Lee et al., 2009
Prostate cancer	Atorvastatin	–	–	–	Reduced PSA levels	Khosropanah et al., 2011
Pancreatic cancer	Combination of Evolocumab, Atorvastatin, Ezetimibe	Early phase I	NCT04862260	Not yet recruiting	Not yet	Chu de Quebec-Universite Laval, 2021

## Conclusion

**FIGURE 1:**
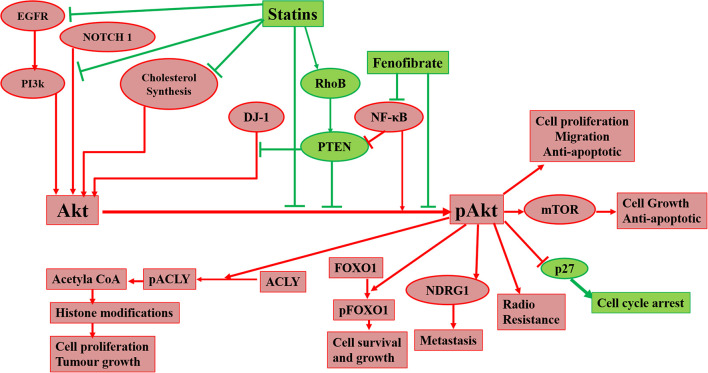
Diagrammatic representation of effects of cholesterol-lowering drugs on Akt in cancer signaling. Statins can act on Akt via inhibition of cholesterol synthesis, EGFR, NOTCH 1, and the promotion of RhoB. Fenofibrate can also act on Akt via inhibition of NF-κB. Inhibition of Akt phosphorylation leads to prevention of radioresistance, cell proliferation, migration, and metastasis of cancer cells and promotion of apoptosis and cell cycle arrest.

Raised cholesterol level is a concern for millions of people as it can lead to a high risk of heart disease. As per WHO, one-third of ischemic heart disease is associated with increased cholesterol levels (World Health Organisation, 2021). Other than heart disease, an increase in cholesterol levels raises the risk of tumorigenesis and elevated cholesterol is one of the characteristics of cancer cells. Cholesterol-lowering drugs have shown promising effects to treat/inhibit a wide range of cancers (Table 2) and our laboratory has also reported the same in previous studies (Ghosh-Choudhury et al., 2010; Mandal et al., 2011; Chowdhury et al., 2017). Among several cholesterol lowering drugs, statins are commonly used to control cholesterol levels and have been extensively studied to check anti-tumorigenic effects. Cholesterol-lowering drugs affect different signaling pathways in cancer cells (Figure 1). We had earlier noticed that anti-diabetic drug metformin and *N*-arachidonoyl dopamine inhibit breast cancer growth and epithelial to mesenchymal transition by decreasing cholesterol content in cancer cells (Sharma et al., 2019; Bandyopadhayaya et al., 2021). Akt is one of the most common signaling pathways in cancer cells for cell survival, angiogenesis, and tumorigenesis. It increases glucose metabolism and promotes lipogenesis. It promotes the SREBP, one of the critical regulators for cholesterol synthesis and a target of statins. The effect of cholesterol-lowering drugs via Akt signaling has been reported in several cancer types, but need to be further extended. These drugs can modulate several Akt pathways and show the anti-tumorigenic effects. These drugs can inhibit survivin to induce apoptosis and can radio sensitize the cancer cells by inhibiting Akt signaling. They overcome the resistance of cancer cells against the cytotoxic drugs via Akt inhibition. All these aspects manifest the importance of Akt as a key target of cholesterol-lowering drugs to inhibit tumorigenesis. Indeed, activation of NF-kB which is a key downstream target of Akt is also responsible for the development of chemoresistance. Our study revealed that cholesterol lowering simvastatin inhibits NF-kB by targeting PTEN/Akt signaling to attenuate cancer growth (Ghosh-Choudhury et al., 2010). Our other studies documented that omega-3 fatty acids target PI3K/Akt/NF-kB axis, and inhibit cancer growth and metastasis in breast cancer models (Ghosh-Choudhury et al., 2009; Mandal et al., 2010, 2012). Thus, the combination of statins and omega-3 fatty acids might work effectively in controlling cancer growth in chemoresistance cells. Despite these studies, there is a requirement to study the effect of inhibition of Akt signaling by different categories of cholesterol-lowering drugs on tumorigenesis as these studies are still limitedly done. Not only this, these studies are further required to expand to other cancers so that an overall picture of the effects of these drugs on Akt signaling could be drawn.

**TABLE 2:** Anti-tumorigenic effects of cholesterol-lowering drugs by targeting Akt and associated molecules.

S.N.	Cancer type	Cholesterol-lowering drug	Molecule/pathway target	Effect on cancer cells	Reference
(1)	Lung cancer	Simvastatin	Akt; Survivin; PARP	↑Apoptosis	Hwang et al., 2011
			Akt	↑Apoptosis	Kim et al., 2015
		Lovastatin	PI3k/Akt; ACLY	↑Anti-tumor	Hanai et al., 2012
			EGFR-PI3k-Akt; AMPK	↑Apoptosis ↑Radiation sensitization	Sanli et al., 2011
		Fluvastatin	Akt; Braf/MEK/ERK1/2	↓Growth ↑Apoptosis	Zhang et al., 2019
(2)	Breast cancer	Atorvastatin	p-Akt	↓Growth	Beckwitt et al., 2018
			PTEN/Akt; RhoB	↓Proliferation, ↓Migration, ↑Apoptosis	Ma et al., 2019
		Simvastatin	PI3K/Akt/mTOR	↑Apoptosis, ↓Proliferation	Wang et al., 2016
			PTEN/Akt	↓Cell viability	Park et al., 2013
			PTEN/Akt; NFκB	↓Growth	Ghosh-Choudhury et al., 2010
		Lovastatin	PTEN/Akt; DJ-1; NDRG1	↓Cell viability ↓Metastasis	Klawitter et al., 2010
		Fenofibrate	Akt/NF-κB	↑Apoptosis	Sun et al., 2019
(3)	Colorectal cancer	Lovastatin	Akt	↑Cytotoxicity	Mantha et al., 2005
		Atorvastatin	Akt; PTEN; PI3k; PDK1	↑Apoptosis ↓Proliferation	Xiao et al., 2008
(4)	Prostate cancer	Simvastatin; fluvastatin	Akt/FOXO	↑Apoptosis	Deng et al., 2019
		Atorvastatin	Akt; cholesterol synthesis	↓Cell survival	Chen X. et al., 2016
(5)	Liver cancer	Atorvastatin	Akt; mTOR; GSK3β	↓Proliferation	Roudier et al., 2006
		Simvastatin	Akt;	↑Apoptosis	Huang et al., 2015
		Fenofibrate	Akt	↓Proliferation	Yamasaki et al., 2011
(6)	Pancreatic cancer	Atorvastatin	Akt and its downstream targets	↓Proliferation ↑Apoptosis	Mistafa and Stenius, 2009
			PI3/Akt	↓Progression	Mohammed et al., 2012
			Akt-ACLY	↓Proliferation ↓Growth	Carrer et al., 2019
(7)	Oral cancer	Lovastatin	EGFR; PI3k/Akt	↓Proliferation ↓Cell survival	Zhao et al., 2010
		Fenofibrate	Akt/mTOR	↓Progression	Jan et al., 2016
(8)	Leukemia	Statins (atorvastatin, lovastatin, and simvastatin)	Akt/mTOR/p70S6K	↑Autophagy	Vilimanovich et al., 2015
		Fluvastatin and simvastatin	Akt	↓Proliferation ↑Apoptosis	Wang J.J. et al., 2018
		Simvastatin	Akt	↓Proliferation ↑Apoptosis	Zeng et al., 2012
		Lovastatin	Ras/PI3K/Akt; Ras/Raf/ERK	↓Proliferation ↑Apoptosis	Chen et al., 2015
(9)	Gliomas	Simvastatin	PI3K/Akt/caspase-3	↓Proliferative ↓Migration ↑Apoptosis	Wu et al., 2009

## Author Contributions

NK collected information, prepared the tables and figures, drafted and wrote the manuscript. CCM formulated the study and written the manuscript. Both authors contributed to the article and approved the submitted version.

## Conflict of Interest

The authors declare that the research was conducted in the absence of any commercial or financial relationships that could be construed as a potential conflict of interest.

## Publisher’s Note

All claims expressed in this article are solely those of the authors and do not necessarily represent those of their affiliated organizations, or those of the publisher, the editors and the reviewers. Any product that may be evaluated in this article, or claim that may be made by its manufacturer, is not guaranteed or endorsed by the publisher.
